# ESX-1-induced apoptosis during mycobacterial infection: *to be or not to be, that is the question*

**DOI:** 10.3389/fcimb.2013.00088

**Published:** 2013-12-04

**Authors:** Nacho Aguiló, Dessislava Marinova, Carlos Martín, Julián Pardo

**Affiliations:** ^1^Grupo de Genética de Micobacterias, Department of Microbiología, Medicina Preventiva y Salud Pública, Universidad de ZaragozaZaragoza, Spain; ^2^CIBER Enfermedades Respiratorias, Instituto de Salud Carlos IIIMadrid, Spain; ^3^Cell Immunity in Cancer, Inflammation and Infection group, Biomedical Research Centre of Aragon, Nanoscience Institute of Aragon, Aragon I+D Foundation, IIS Aragon/University of ZaragozaZaragoza, Spain

**Keywords:** *Mycobacterium tuberculosis*, apoptosis, virulence, necrosis, attenuated strains, cell death

## Abstract

The major *Mycobacterium tuberculosis* virulence factor ESAT-6 exported by the ESX-1 secretion system has been described as a pro-apoptotic factor by several independent groups in recent years, sustaining a role for apoptosis in *M.* tuberculosis pathogenesis. This role has been supported by independent studies in which apoptosis has been shown as a hallmark feature in human and mouse lungs infected with virulent strains. Nevertheless, the role of apoptosis during mycobacterial infection is subject to an intense debate. Several works maintain that apoptosis is more evident with attenuated strains, whereas virulent mycobacteria tend to inhibit this process, suggesting that apoptosis induction may be a host mechanism to control infection. In this review, we summarize the evidences that support the involvement of ESX-1-induced apoptosis in virulence, intending to provide a rational treatise for the role of programmed cell death during *M. tuberculosis* infection.

## Introduction

Host cells can recognize any self-damage (aging, pathogen infection, DNA damage, etc.) and as a result can activate the extrinsic or the intrinsic apoptotic program that leads to programmed cell death. This process is regulated by a family of cysteine proteases (caspases) (Hotchkiss and Nicholson, 2006), the Bcl-2 family proteins consisting of pro-apoptotic (Bid, Bak, Bax, Bim, PUMA, etc) and anti-apoptotic (Bcl-2, Bcl-XL, Mcl-1, etc) members (Adams and Cory, 2007) and the p53 family (p53, p63, and p73) involved in cell cycle control and induction of apoptosis following DNA damage (Levrero et al., 2000). The major effect of apoptosis is the generation of specific signals to attract phagocytes to remove dying cells and avoid tissue damage (Ravichandran, 2011). Resultantly, for some time apoptosis was considered a silent form of cell death. However, in recent years, it has become evident that apoptotic cell death is not always silent, but can induce activation of the immune system against tumoral or pathogen-derived antigens contained within dying cells (Kono and Rock, 2008) by a process known as cross-priming (Bevan, 1976). This process involves the activation of MHC-I restricted naive CD8^+^ T cells by dendritic cells that have engulfed exogenous antigens (den Haan et al., 2000) including dying cells (Ronchetti et al., 1999).

Intracellular pathogens can modulate programmed cell death by blocking or promoting host cell apoptosis to favor infection outcome (Finlay and McFadden, 2006). Some intracellular pathogens such as *Salmonella* (Guiney, 2005), *Shigella* (Zychlinsky et al., 1992), or *Yersinia* (Monack and Falkow, 2000) use apoptosis as a colonization mechanism to infect new host cells, thus, avoiding exposure to extracellular host defence mechanisms (Finlay and McFadden, 2006). The different lifestyle and replication adaptation of obligate vs. facultative intracellular pathogens could explain the paradox of programmed cell-death modulation by different intracellular pathogens. Moreover, the same microorganism can inhibit or induce apoptosis depending on the stage of infection, as described in the case of Chlamydia (Byrne and Ojcius, 2004). In continuation, we summarize the experimental evidences supporting either inhibition or activation of apoptosis as mechanisms of *M. tuberculosis* virulence with the aim to provide a rational explanation of how apoptosis modulation can affect mycobacterial pathogenesis.

## ESX-1 dependent apoptosis

The role of apoptosis in *M. tuberculosis* infection has been a matter of intense debate over the last years. Conflicting results supporting either inhibition (Balcewicz-Sablinska et al., 1998; Keane et al., 2000; Chen et al., 2006; Gan et al., 2008; Divangahi et al., 2009; Behar et al., 2010) or induction (Rojas et al., 1997; Schaible et al., 2003; Gao et al., 2004; Derrick and Morris, 2007; Leong et al., 2008; Davis and Ramakrishnan, 2009; Seimon et al., 2010; Aporta et al., 2012) of apoptosis as a virulence strategy to establish and spread mycobacterial infection have been reported.

One of the strongest experimental findings supporting the ability of virulent *M. tuberculosis* to induce apoptosis in host macrophages is the expression of the major virulence factor 6 kDa early secretory antigenic target (ESAT-6), secreted through the ESX-1 export system. Different groups have independently reported that ESAT-6 secretion is essential for apoptosis induction on infected cells (Derrick and Morris, 2007; Choi et al., 2010; Aporta et al., 2012; Aguilo et al., 2013). Moreover, provided that loss of ESAT-6 is linked to attenuation of different mycobacterial strains (Pym et al., 2002), it is tempting to speculate that ESAT-6-induced cell death could represent a viable mechanism of virulence for *M. tuberculosis*. Attenuated mycobacterial strains, like BCG or the live-attenuated *phoP-/*DIM-deficient *M. tuberculosis* strain MTBVAC (Arbues et al., 2013), which lack a functional ESX-1 system have lost the ability to induce apoptosis and cell death (Rojas et al., 1997; Schaible et al., 2003; Aporta et al., 2012; Aguilo et al., 2013). Indeed, Winau et al induced apoptosis externally on BCG-infected macrophages by serum deprivation to demonstrate that apoptosis is linked to cross-priming of mycobacterial antigen-specific CD8+ T-cells (Winau et al., 2006). Remarkably, RD1-complemented BCG, which fully restores ESAT-6 secretion and virulence, results highly pro-apoptotic *in vitro* and *in vivo* (Aguilo et al., 2013).

## How can apoptosis contribute to virulence?

Data from different works provide evidence that ESX-1-induced apoptosis can contribute to virulence by spreading infection. RD1-defficient H37Rv, which is unable to trigger apoptosis (Derrick and Morris, 2007), has shown impaired capacity to colonize new uninfected cells (Gao et al., 2004; Guinn et al., 2004), suggesting that apoptosis favors cell-to-cell bacterial spread. Confirming the role of apoptosis in host colonization by virulent mycobacteria, we recently reported that *in vitro* apoptosis induction by several virulent strains promotes bacterial spread into bystander macrophages. Conversely, ESX-1-deficient strains have lost cell-to-cell colonization capacity, indicating that this mechanism is dependent on ESAT-6 secretion (Aguilo et al., 2013). Confirming these data *in vivo*, the importance of ESX-1 dependent apoptosis for bacterial spread has been shown in the Zebra fish model (Davis and Ramakrishnan, 2009).

Supporting the hypothesis of apoptosis induction as an advantageous cell-to-cell spread mechanism for pathogenic mycobacteria, Schaible et al showed that virulent *M. tuberculosis* Erdman strain induces apoptosis in both macrophages and dendritic cells and cell death is accompanied by the generation of typical apoptotic bodies (Schaible et al., 2003). In a series of elegantly controlled experiments the authors showed that these apoptotic bodies were engulfed by bystander macrophages using classical phagocytic receptors for apoptotic cells.

## How does ESAT-6 induce apoptosis on the host cell?

Previous works suggest that endoplasmic reticulum (ER)-stress associated pathways are activated and induce apoptosis during *M. tuberculosis* infection in an ESAT-6-dependent fashion (Choi et al., 2010; Grover and Izzo, 2012). Lim et al. (2011) demonstrated the activation of classical ER-stress markers in macrophages during *M. tuberculosis* infection *in vitro*. Co-localization of ER-stress and apoptotic markers has also been found in both mouse and human infected lungs indicating that these signaling routes are activated by *M. tuberculosis* under physiological conditions (Seimon et al., 2010).

Different intracellular events can trigger activation of ER-stress associated pathways leading to the activation of the intrinsic apoptotic pathway (Gorlach et al., 2006). ESAT-6 has been reported to increase intracellular Ca^2+^ concentration and reactive oxygen species (ROS) (Choi et al., 2010), which are classical ER-stress activators. A mechanism involving ER-stress and ROS induction has been described for *M. kansasii*-induced apoptosis (Lim et al., 2013). Interestingly, RD1-deficient H37Rv mutant is unable to cause intracellular Ca^2+^ increment and subsequent calpain activation (Yang et al., 2013), suggesting that ESAT-6 is responsible for triggering the initial events that would lead to cell death through ER-stress. One of the main downstream regulators of ER-stress-induced apoptosis is the ASK1-p38MAPK route (Matsuzawa et al., 2002). ASK1-deficient macrophages are not able to phosphorylate p38MAPK after *M. tuberculosis* infection and as a consequence are highly resistant to apoptosis induced by *M. tuberculosis* (Kundu et al., 2009). Additionally, p38MAPK inhibition has also been described to profoundly abrogate *M. tuberculosis*-induced apoptosis (Aleman et al., 2004; Kundu et al., 2009; Aguilo et al., 2013).

Ultimately, the mitochondrial apoptotic pathway is activated in *M. tuberculosis*-infected macrophages involving the release of cytochrome *c* (Abarca-Rojano et al., 2003; Chen et al., 2006) and the subsequent activation of caspases 9 and 3 (Uchiyama et al., 2007; Aporta et al., 2012; Lim et al., 2013). Accordingly, inhibition of caspase 9, which is the initiating caspase of the intrinsic apoptotic pathway, impairs *M. tuberculosis*-induced apoptosis Martin et al. ASK1-induced cell death has also been described to depend on the activation of mitochondrial apoptotic pathway (Hatai et al., 2000), possibly linking ER stress induced by virulent *M. tuberculosis* with the activation of the intrinsic apoptotic pathway.

In addition to its ability to directly induce apoptosis, interaction of ESAT-6 with the host cell has been shown to interfere with different signaling cascades, such as the inflammatory NF-κ B pathway (Pathak et al., 2007) and autophagy (Romagnoli et al., 2012). Remarkably, these pathways are naturally associated with cell survival and it is possible that by interfering with them, ESAT-6 could be sensitizing cells to undergo programmed cell death by down-modulating anti-apoptotic cellular mechanisms. A similar mechanism has been described for other microorganisms, such as *Yersinia*, where virulence factor YopJ abrogates MAPK and NF-κ B to favor apoptosis induction (Zhang et al., 2005).

Importantly, different groups have found that ESAT-6 possesses pore-forming and membrane lysing capacities (de Jonge et al., 2007; Smith et al., 2008). Thus, virulent mycobacteria can cause phagosome membrane disruption in an ESAT-6-dependent fashion, reaching the cytosol and triggering cell death (van der Wel et al., 2007; Houben et al., 2012; Simeone et al., 2012). Consequently, host macrophage death is concurrent with contact of *M. tuberculosis* with the cytosol, suggesting that *M. tuberculosis* needs to physically reach the cytosol to trigger the pro-apoptotic signaling cascade.

## *M. tuberculosis* inhibits apoptosis and promotes necrosis

In discrepancy with data discussed above, different groups maintain that the ability to trigger apoptosis is more evident in attenuated strains, while virulent mycobacteria tend to inhibit this process (Keane et al., 1997, 2000; Balcewicz-Sablinska et al., 1998; Danelishvili et al., 2003; Hinchey et al., 2007), sustaining a role for apoptosis induction as a host mechanism to control infection Martin et al., rather than a virulence mechanism of infectivity. Several works indicate that TNFα is the main inductor of apoptosis by attenuated strains (Keane et al., 1997; Balcewicz-Sablinska et al., 1998). Some anti-apoptotic genes (e.g., *nuoG*) have been described to exert their function by inhibiting TNFα secretion (Miller et al., 2010). Conversely, virulent strains would promote necrotic-like cell death, which would allow bacteria to be released to the extracellular milieu, restarting the cycle of re-infection (Chen et al., 2006; Gan et al., 2008; Lee et al., 2011). Virulent *M. tuberculosis* has been reported to favor necrotic cell death by interfering with the plasma membrane repair mechanisms (Divangahi et al., 2009) thus, blocking the synthesis of prostaglandin E_2_ (PGE_2_), which is important for lysosome-dependent membrane repair (Divangahi et al., 2010).

A possible reason for the discrepancies regarding the cell death phenotype induced by *M. tuberculosis* could lie in that most of the studies in this field have been conducted under *in vitro* conditions, where the use of a single procedure to differentiate between apoptotic and necrotic phenotype is common. Nevertheless, the use of parallel methodologies to accurately define a cell death phenotype is recommended (Galluzzi et al., 2009). In this regard, some works that show virulent *M. tuberculosis* to induce necrosis have reported the appearance of typical apoptotic features, such as DNA fragmentation and nuclear fragmentation and/or condensation (Lee et al., 2011) in addition to plasma membrane permeability, a necrotic cell death characteristic (Butler et al., 2012). A common methodology to discern apoptosis from necrosis is to measure phosphatidylserine exposure together with plasma membrane integrity. This procedure can result confusing since under *in vitro* conditions the appearance of secondary necrosis is usual in cells which might have undergone apoptosis at earlier time points (Krysko et al., 2008). Finally, different experimental procedures and absence of standardized protocols could contribute to varying and discrepant results. For example, in the same experimental design, apoptotic- or necrotic-like phenotype can be observed depending on whether low or high multiplicity of infection (MOI) is used, respectively (Aporta et al., 2012). These observations indicate that the type of cell death induced by *M. tuberculosis in vitro* can vary depending on the experimental conditions. As such, it is difficult to define an absolute cell death phenotype *in vitro* that can be extrapolated to what would be observed under real physiological situations.

## Lessons from *in vivo* data

Based on *in vitro* results, several authors have attributed bactericidal properties to mycobacteria-induced apoptosis (Lee et al., 2006; Martin et al., 2012) in a process that depends on efferocytosis, where phagocytosed mycobacteria contained within efferosomes are unable to arrest phagosome acidification leading to loss of bacterial viability (Martin et al., 2012). However, experiments *in vivo* with an *M. tuberculosis nuoG* mutant, characterized by an enhanced capacity to induce apoptosis in mouse lungs, did not show loss of viability of the mutant as compared to wild-type strain following low-dose aerosol infection in mice (Blomgran et al., 2012). These data suggest that the described bactericidal capacity of *M. tuberculosis-*induced apoptosis *in vitro* is not observed *in vivo* and more importantly, they indicate that enhancing the pro-apoptotic potential of a virulent strain does not reduce its virulence in a physiological infection.

Unlike discrepant *in vitro* results, *in vivo* data seem to be more consensual. Different independent works have shown the presence of apoptotic markers such as active caspase 3 or TUNEL in murine and human lungs following virulent mycobacterial infection (Keane et al., 1997; Klingler et al., 1997; Leong et al., 2008; Seimon et al., 2010; Aporta et al., 2012; Blomgran et al., 2012; Aguilo et al., 2013). In the zebra fish model of piscine tuberculosis, *M. marinum* triggers apoptosis in an ESAT-6-dependent manner as a spread mechanism of infection (Davis and Ramakrishnan, 2009). Supporting an *in vivo* role for apoptosis in cell-to-cell bacterial spread, Blomgran et al showed that pulmonary infection of mice with the *nuoG* mutant correlates increased apoptosis induction with a higher cell-to-cell transmission capacity as compared to wild-type strain (Blomgran et al., 2012). Conversely, attenuated strains BCG and MTBVAC, with a defective ESX-1 system, do not trigger apoptosis in lungs of mice (Aporta et al., 2012; Aguilo et al., 2013). Despite discrepant data on the role of mycobacteria-associated apoptosis *in vitro*, these results suggest that *in vivo* apoptosis is a feature associated with ESAT-6 secretion and virulence.

Different authors have suggested that apoptosis is a host defence mechanism as it is an effective cross-priming inducer, favoring cross-presentation of mycobacterial antigens contained in apoptotic bodies in the local lymph nodes (Schaible et al., 2003; Winau et al., 2006; Hinchey et al., 2007; Divangahi et al., 2010; Blomgran et al., 2012). These data could be in apparent discrepancy with the possible role of apoptosis as a virulence mechanism used by mycobacteria to favor cell-to-cell spread. Nonetheless, virulent *M. tuberculosis* strains, shown to trigger apoptosis *in vivo*, also elicit a strong specific immune response (Cooper, 2009) indicating that these two events are not necessarily exclusive. Indeed, data from experimental mouse models indicate that one of the best strategies of *M. tuberculosis* to successfully colonize the host is to delay the establishment of an effective adaptive immune response during the early phases of infection (Cooper, 2009). It is estimated that the adaptive response takes around 2–3 weeks to be triggered in the local lymph nodes and to migrate to the lungs (Wolf et al., 2008). This is enough time for *M. tuberculosis* to replicate without host resistance, allowing mycobacteria to reach critical bacterial burden against which the adaptive immune response could only exert a bacteriostatic effect (Cooper, 2009). Hence, if mycobacteria-loaded apoptotic bodies must migrate to the lymph nodes for cross-presentation (Winau et al., 2006), this would imply valuable time before the host could establish an effective response in the early stages to control the infection. During this critical period, *M. tuberculosis* would induce apoptosis in host phagocytes allowing bacterial spread and gain of new replication niches, while maintaining the intracellular environment. In line with this model, mouse infection with the highly apoptogenic *M. tuberculosis nuoG* mutant showed higher cell-to-cell spread capacity together with increased efficiency to trigger specific adaptive immune response as compared to wild-type *M. tuberculosis* (Blomgran et al., 2012).

**Figure 1:**
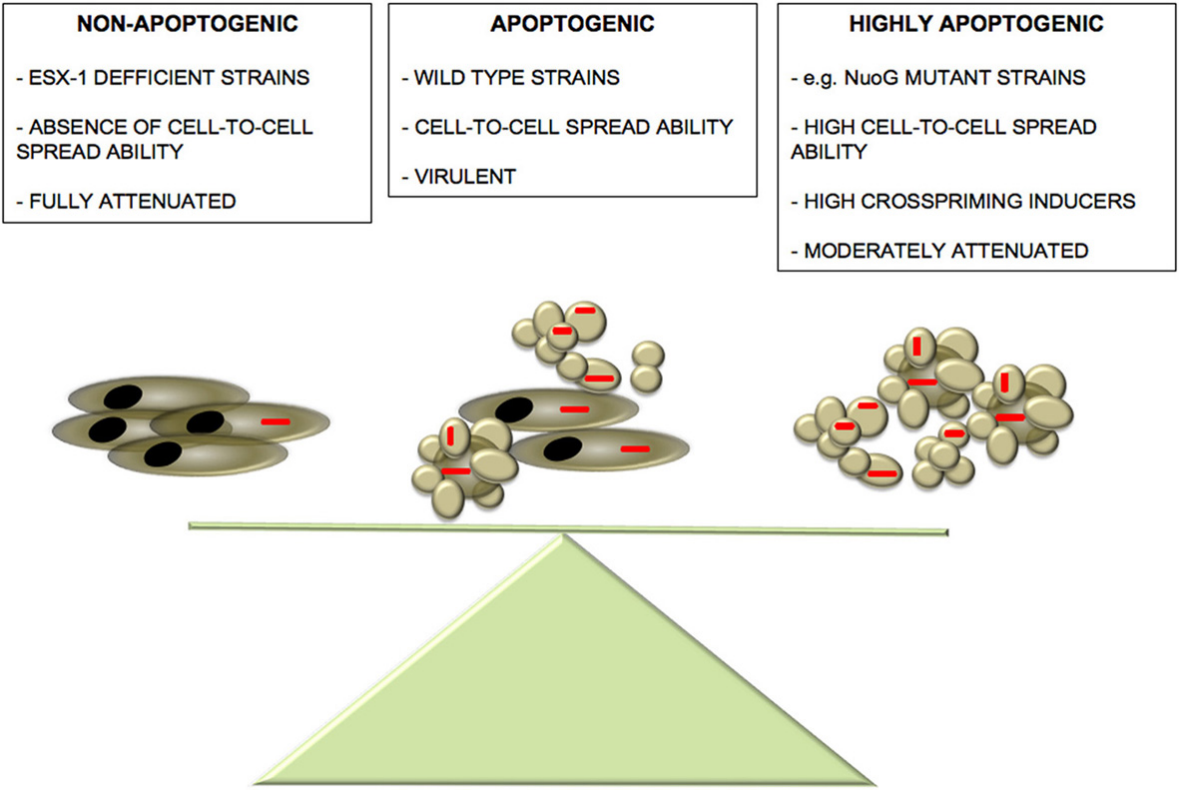
**Balance between *M. tuberculosis*-induced apoptosis and virulence**. We propose the existence of a delicate balance between mycobacteria-induced apoptosis and pathogenesis. Absence of apoptosis observed in ESX-1-deficient strains leads to a sound attenuation phenotype accompanied by abrogated cell-to-cell spread capacity (Aguilo et al., 2013). On the other hand, although highly apoptogenic strains show an increased cell-to-cell spread capacity compared to wild-type strains, they might present higher visibility to the host favoring an accelerated establishment of host immune defences, thus, tipping the balance to the favor of the host. In the “golden mean” between the two extremes of apoptosis induction, virulent wild-type strains have active both pro- and anti-apoptotic mechanisms that even though partially limit cell-to-cell infectivity, they impair the rapid establishment of host immune response, thus, favoring *M. tuberculosis* pathogenesis.

Thenceforth, which could be the physiological significance of cross-priming of specific T-cell responses by apoptotic bodies during *M. tuberculosis* infection? We speculate that apoptosis could have dual and opposing roles during the interaction of mycobacteria with the host. The pathogen could favor cell-to-cell bacterial spread at early stages, as well as induction of mycobacteria-specific host immune response, a process that would be accelerated if the levels of apoptosis were excessive. This way, during co-evolution with the host, *M. tuberculosis* could have developed pro-apoptotic ESX-1-dependent mechanisms essential for successful cell-to-cell infection spread and in parallel, molecular mechanisms (e.g., *nuoG, secA2*) to restrict excessive apoptosis that would otherwise result in an accelerated generation of host immunity that could impair propagation of infection in the lungs. (Hinchey et al., 2007; Blomgran et al., 2012). This hypothesis is summarized in Figure 1.

## Concluding remarks

**Figure 2:**
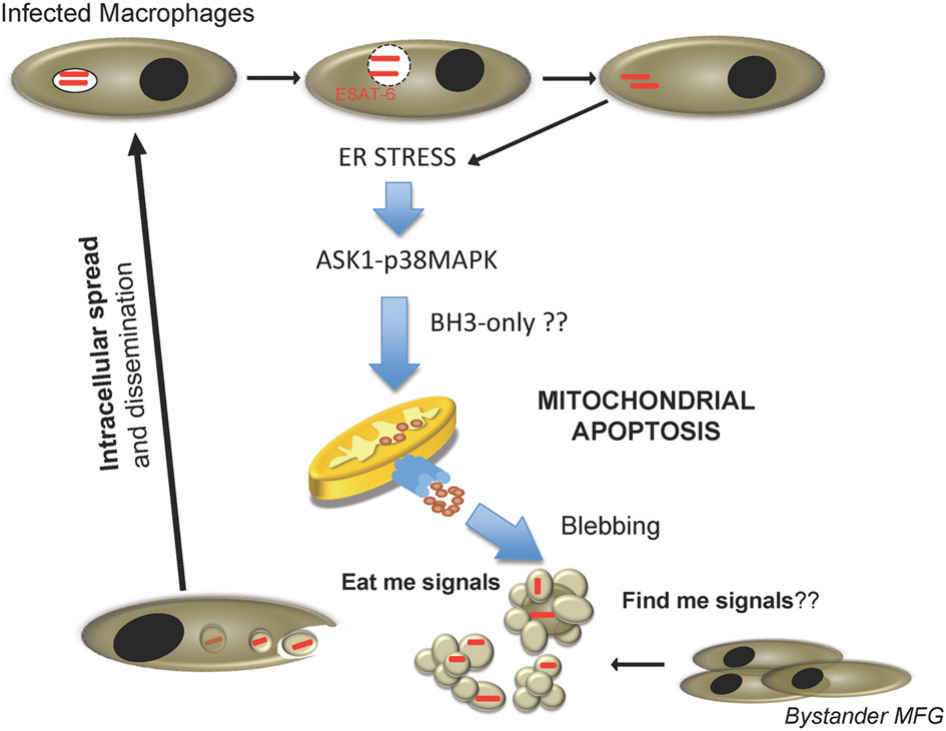
**Proposed cell-to-cell spread model for *M. tuberculosis* during host cell infection**. Internalized *M. tuberculosis* disrupts phagosome in an ESAT-6 dependent manner. After reaching the cytosol, mycobacteria trigger ER stress, and activate pro-apoptotic cellular routes involving the ASK1-p38MAPK signaling axis, and causing host cell apoptosis by activation of the mitochondrial apoptotic pathway. Consequently, fresh macrophages are recruited and *M. tuberculosis*-containing apoptotic bodies are phagocytosed by new cells, re-starting the infection cycle.

Can apoptosis and necrosis be mutually exclusive processes in the context of *M. tuberculosis* infection? If we consider only the available *in vitro* data in the literature, the answer to this question seems to be affirmative. However, existing *in vivo* data suggests that both processes can occur during *M. tuberculosis* infection in different spatiotemporal stages. Data indicate that apoptosis is a common feature associated with virulent strains crucial to promote dissemination and host colonization. Thus, ESX-1-mediated apoptosis could be a critical step during the early stages of host-pathogen interaction, when bacterial load is low and few macrophages are infected. *M. tuberculosis* contained within apoptotic bodies would recruit and infect bystander macrophages, allowing infection of new host cells while maintaining an intracellular environment (Figure 2). *M. tuberculosis* is a successful intracellular pathogen, which in its co-evolution with the human host has developed multiple effective mechanisms to prevent intracellular defences. In this context, little evidence exists for mycobacterial strategies targeting extracellular antimicrobial barriers. Apoptosis induction could allow mycobacteria to propagate in the absence of inflammatory reactions normally associated with release of cytosolic material extracellularly, a typical feature of necrotic cell death. Efferocytosis of apoptotic bodies by bystander macrophages has been shown to create an anti-inflammatory environment due to IL-10 and PGE2 release known to inhibit macrophage function which could contribute to delayed establishment of the adaptive immune response (Medeiros et al., 2009).

**Figure 3:**
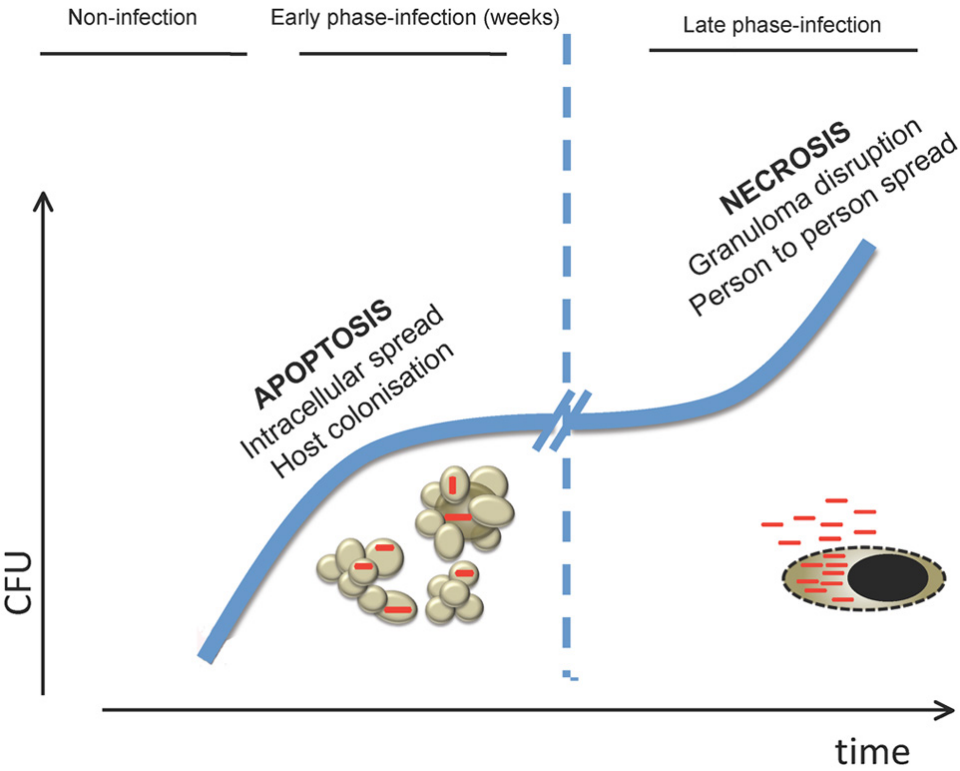
**Model of how cell death is involved in the different stages of *M. tuberculosis* host infection**. In the early stages of infection, killing of macrophages by apoptosis would allow *M. tuberculosis* to spread cell-to-cell in the lung in the absence of inflammatory mediators associated with necrotic release of intracellular content. This would permit bacteria to replicate in the preferred intracellular environment prior to establishment of an effective adaptive immune response. In the latest stages of infection, when bacterial burden is high, *M. tuberculosis* would induce necrosis on infected cells leading to mycobacterial release to the extracellular medium. As a consequence, granuloma is disrupted allowing aerosol transmission to new hosts.

Conversely, during active tuberculosis disease high bacterial burden would induce massive necrosis in host cells breaking the granuloma and reaching the respiratory tract to infect new individuals. Macrophages infected with high MOIs have been shown to die in a necrotic-like way (Lee et al., 2006). Probably, an exacerbated immune response also participates in this process. A model proposed in zebra fish indicates that during *M. marinum* infection, high levels of TNFα production lead to necroptosis events (Roca and Ramakrishnan, 2013). Indeed, caseation and necrosis are usual events observed in granulomas *in vivo*. It is possible that depending on the different environments encountered during the various phases of infection, *M. tuberculosis* is able to modulate the way that the host cell dies, favoring a successful infection and disease outcome (Figure 3).

The debate about whether apoptosis is beneficial for the bacteria or the host during mycobacterial infection remains open. Unlike *in vitro* observations, which tend to attribute an only role to apoptosis in mycobacterial pathogenesis, *in vivo* data seem to indicate that the answer to this question is neither black nor white. The available experimental evidence indicates that mutant strains without a functional ESX-1 system, which are not able to induce apoptosis/cell death, are much more attenuated than mutants in which apoptosis is enhanced (e.g., by deletion of *nuoG*), suggesting that ESX-1-mediated apoptosis is eminently a virulence mechanism that favors cell-to-cell mycobacterial spread and host colonization. Nevertheless, excessive apoptosis induction could result beneficial for the host as cross-priming is favored. Accordingly, it seems that apoptosis could have dual and opposing roles during infection where both the host and the pathogen attempt to use this process to tip the balance to their benefit. Thus, the fundamental question *to be or not to be* during mycobacterial infection results highly complex and does not seem to have a single answer.

### Conflict of interest statement

The authors declare that the research was conducted in the absence of any commercial or financial relationships that could be construed as a potential conflict of interest.
